# Management challenges of traumatic spondylolisthesis of the Axis with an unusual C2-C3 posterior subluxation in a paediatric patient: case report and literature review

**DOI:** 10.4314/ahs.v18i2.31

**Published:** 2018-06

**Authors:** Kaunda Ibebuike, Mark Roussot, James Watt, Robert Dunn

**Affiliations:** 1 Visiting Spine Fellow, Division of Neurosurgery, Department of Surgery, Imo State University Teaching Hospital, Orlu, Nigeria; 2 Department of Orthopaedic Surgery, Groote Schuur Hospital, Cape Town, South Africa

**Keywords:** Management challenges, traumatic spondylolisthesis of the axis, C2-C3 subluxation, paediatric, anterior cervical discectomy and fusion (ACDF)

## Abstract

**Introduction:**

Paediatric cervical spine injuries are uncommon. Traumatic spondylolisthesis of the axis (TSA) is commonly encountered in the trauma setting. The management of TSA may be surgical or non-surgical. Decision making is quite challenging depending on patient presentation and nature of injury, and even more so in the paediatric age group.

**Objectives:**

To present a case report highlighting the challenges in the management of TSA.

**Methods:**

We present an 8 year old male, who sustained a bilateral C2 pars fracture with associated unusual C2-C3 posterior subluxation.

**Results:**

Neuroradiological studies identified the fracture/subluxation of C2-C3 and revealed an intact but posteriorly displaced C2-C3 disc causing cord compression. An Extension Halter traction was initially commenced. This seemed to have worsened the patient's neck pains, and caused motor weakness and autonomic dysfunction. An anterior cervical discectomy and fusion was finally decided on and performed after evaluation and brainstorming by our spinal Unit. Intra-operative findings revealed separation of the C2-C3 disc from the C3 superior end plate which probably explains the unusual nature of the subluxation.

**Conclusion:**

The case shows that surgical intervention as a primary management for TSA even in the paediatric age group is safe and also avoids risks inherent in conservative management.

## Introduction

Traumatic spondylolisthesis of the axis (TSA) is defined as bilateral fractures of the pars inter-articularis.^1^,^2^ This injury has been termed “Hangman's fracture” due to its similarity to that seen following judicial hangings.^1^ TSA is a common injury of the upper cervical spine, accounting for 20% – 22% of all axis fractures, and resulting from a combination of cervical hyperextension, compression and rebound flexion, and associated commonly with motor vehicle accidents and falls from a height.^1^,^2^,^3^

In children, cervical spine (C-spine) injuries occur infrequently (1% to 2%); and yet may lead to significant disability and even death.^4^–^8^ When these occur, the impact may be devastating emotionally, socially and even economically. 5,9,10 More closely, upper pediatric cervical spine injuries are rare (0.6% to 9.5%) in comparison to lower cervical spine injuries.^10^ This therefore presents relatively little exposure as well as a lack of experience in evaluating these injuries in children, unlike in adults where the higher occurrence of traumatic lesions exposes clinicians on a regular basis to adults with potential cervical spine injuries.^11^ This may therefore also affect clinicians' experience in the management of paediatric trauma patients with a potential for these injuries.^11^ Generally the diagnosis and management of cervical spine injury is more complex in children than in adults.^12^ This case illustrates the management challenges faced in the care of an 8-year old child with traumatic bilateral C2 (axis) pars fracture, with associated unusual posterior C2-C3 subluxation and cord compression, and a review of the relevant literature.

## Case report

An 8-year old male was admitted at the Red Cross Children's Hospital, Cape Town, through the Trauma Emergency Department. The patient was brought to the Trauma Resuscitation room in the hospital's casualty by family members with a history of pedestrian vehicle accident. He was unconscious at the scene of the accident but there was no seizures or vomiting. In the resuscitation room, he was found to be breathing spontaneously with an oxygen saturation of 100%. He had a blood pressure of 110/82 mmHg and a pulse rate of 97 beats/min. His Glasgow coma scale (GCS) was 12/15 (E3M5V4) but improved to 15/15 by the following day. He had equal and reactive pupils bilaterally. There were abrasions on the forehead and oozing of blood from the nostrils, and tenderness at the upper part of the back of the neck in the midline, but there was no neurological fallout. A low dose whole body x-ray (LODOX) performed in the trauma resuscitation room revealed an abnormal C2 vertebral body with evidence of subluxation and atlantodens interval (ADI) of 4.9mm (Fig. 1a and 1b).

**Fig. 1a:**
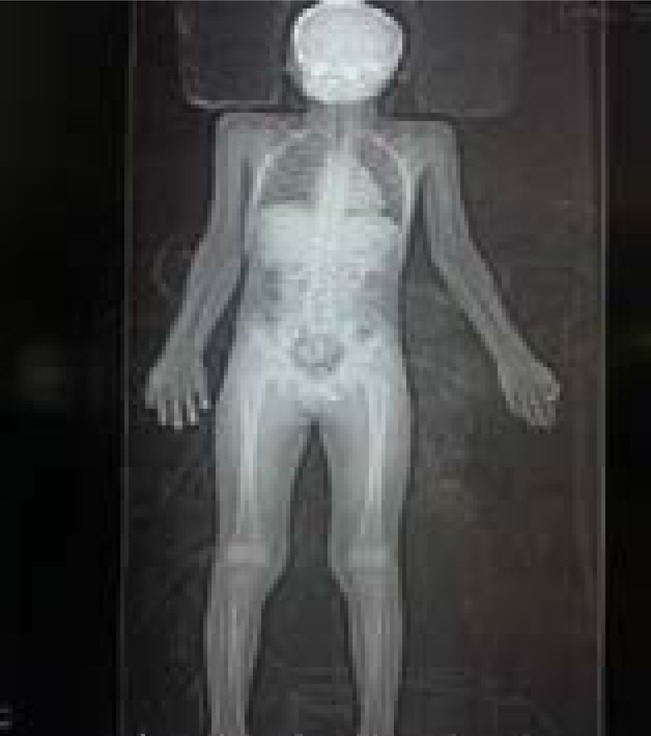
A low dose whole body x-ray (LODOX) performed in the trauma resuscitation room.

**Fig. 1b:**
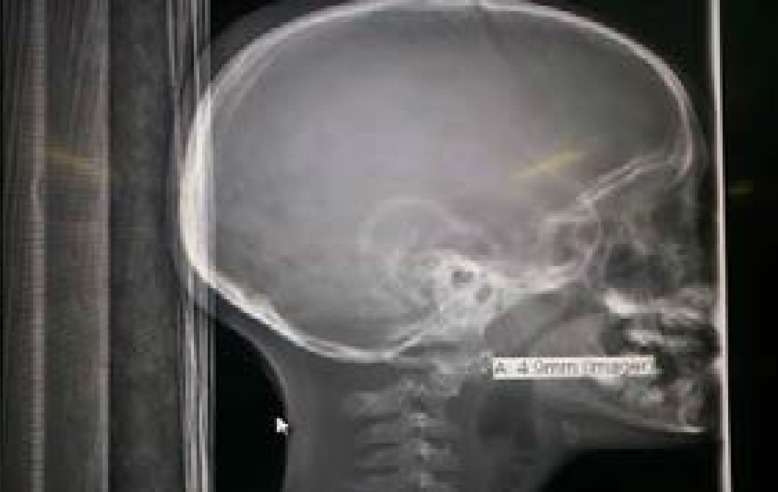
Lateral view of head and C-spine from LODOX showing an abnormal C2 vertebral body with evidence of subluxation.

A CT scan of the brain performed on the same day revealed an essentially normal brain and an old fracture of C2 traversing through the transverse foramina of C2. There was associated posterior subluxation of C2 on C3 with narrowing of the spinal canal at C2/C3 level, but there was no associated prevertebral soft tissue swelling (Fig. 2a and 2b).

**Fig. 2a:**
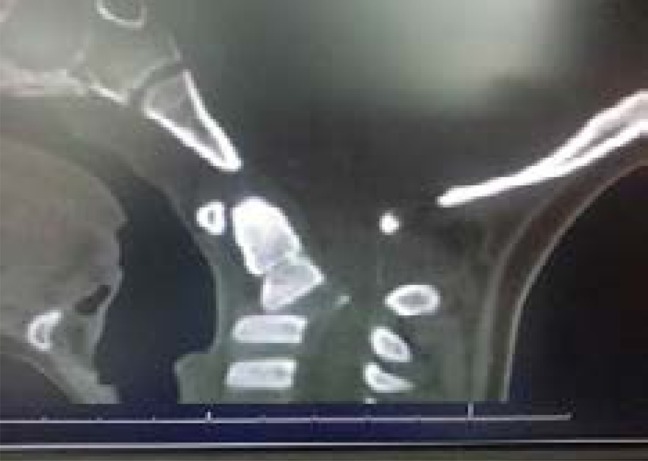
Sagittal CT scan of patient on day of accident/admission showing posterior subluxation of C2 on C3 with narrowing of the spinal canal at C2/C3 level.

**Fig. 2b:**
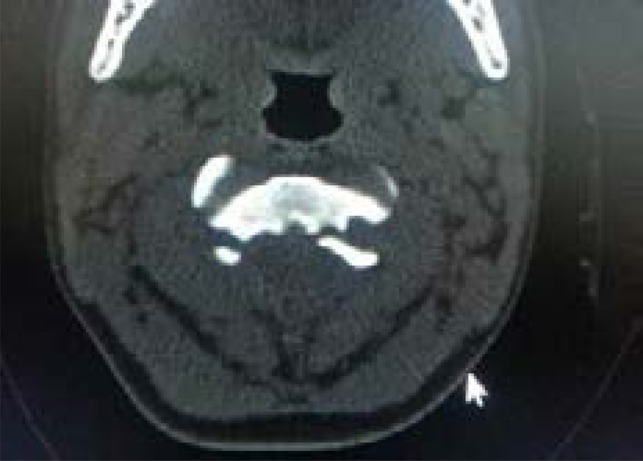
Axial CT scan of patient showing fracture of C2 traversing through the transverse foramina of C2.

**Fig. 3:**
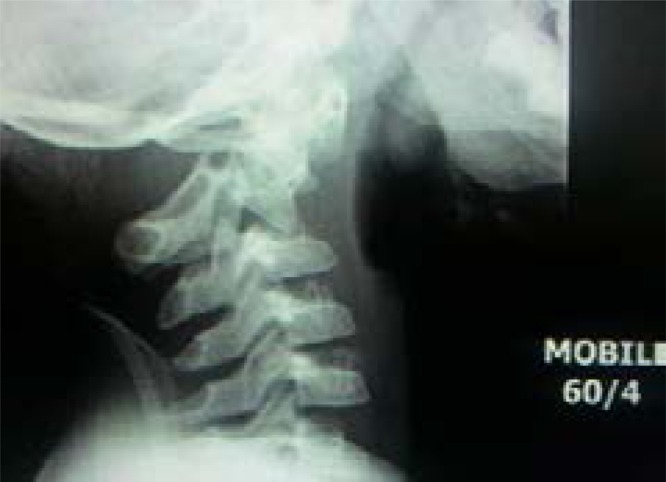
Lateral plain C-spine X-ray showing fractures of bilateral pars interarticularis with subluxation of C2 on C3.

He was subsequently placed on C-spine precautions and soft cervical collar but while on admission in the ward the following day, this was changed to an Extension Halter traction. A follow up plain C-spine X-ray (mobile) performed same day the Halter traction was commenced and with comparison made to the previous LODOX study, revealed significant subluxation of C2 on C3 with fractures of bilateral pars interarticularis (Fig. 3).

However, there was no significant interval change with the LODOX study. The atlantodens interval (ADI) was 3mm and there was no significant prevertebral soft tissue swelling as well. The Halter traction was discontinued and soft cervical collar re-instituted and the head supported by head blocks. On account of persistent exquisitely tender midline upper C-spine, an MRI of the C-spine was performed on the 3rd day post admission and in comparison with the CT study. The MRI identified the fracture/subluxation of C2, and revealed an intact but displaced intervertebral disc between C2 and C3 protruding into the intraspinal canal and causing cord compression. There was associated abnormal high signal intensity extending from C1-C2/C3 intervertebral disc space. Of concern was that the prevertebral soft tissue swelling increased from the CT study and measured 16mm (previously 8mm) and as well showed fluid content (Fig. 4a and 4b).

**Fig. 4a:**
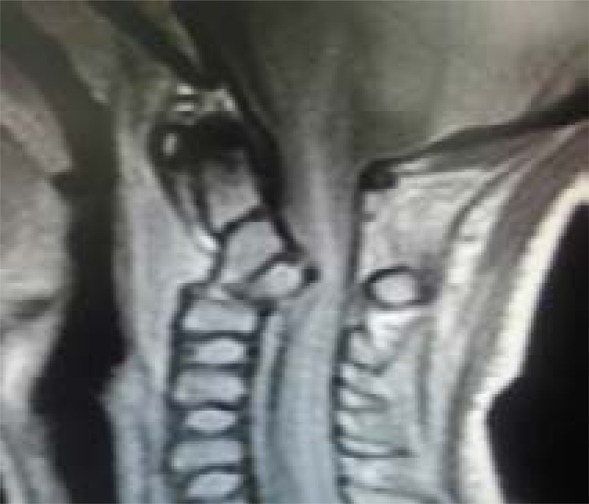
Sagittal cervical proton density weighted (PDW) MRI on day 3 post trauma/admission showing an intact but displaced C2-C3 disc protruding into the intraspinal canal and causing cord compression.

**Fig. 4b:**
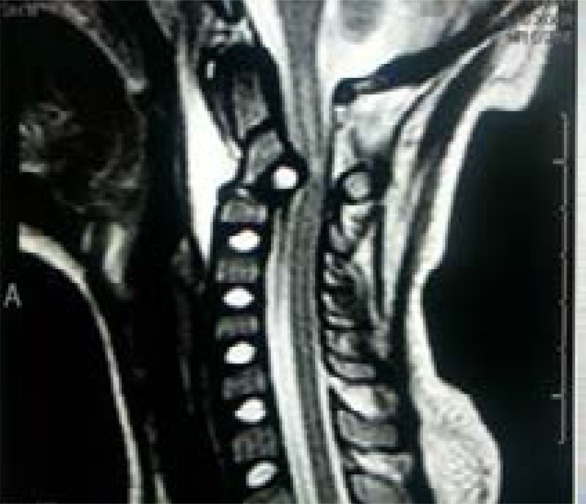
Sagittal cervical T2-weighted MRI showing abnormal high signal intensity extending from C1-C2/C3 disc space and increased prevertebral soft tissue swelling/fluid content.

Given the change in prevertebral soft tissue swelling, the radiologist considered the MRI features to be consistent with acute on chronic C-spine injury with associated cord signal changes. On the 2^nd^ night post admission/injury and after discontinuation of the traction, the patient was noticed to be bradycardic with his pulse rate falling as low as of 40 beats/min, but with stable blood pressure.

He was subsequently admitted in the Paediatric Intensive Care Unit (PICU) to monitor for autonomic dysfunction. A review on the 3^rd^ day post admission by the Spinal Unit revealed decreasing motor power in the upper limbs (4/5 in all muscle groups) and lower limbs (3/5), intact sensations and evidence of upper motor neuron signs. The case was subsequently presented on day 7 post admission at the Spinal Meeting, comprising spine surgeons from the Spinal Unit, Department of Orthopaedic Surgery and Department of Neurosurgery, Groote Schuur Hospital as well as other practicing Spine Surgeons in Cape Town. After extensive deliberations on the pros and cons of conservative versus surgical intervention for this particular traumatic lesion, decision was taken to intervene surgically. On day 10 post admission, a C2-C3 anterior cervical discectomy and fusion (ACDF) using a 4-hole titanum plate/screws and autologous iliac crest bone graft was performed (Fig. 5a, 5b and 6).

**Fig. 5a:**
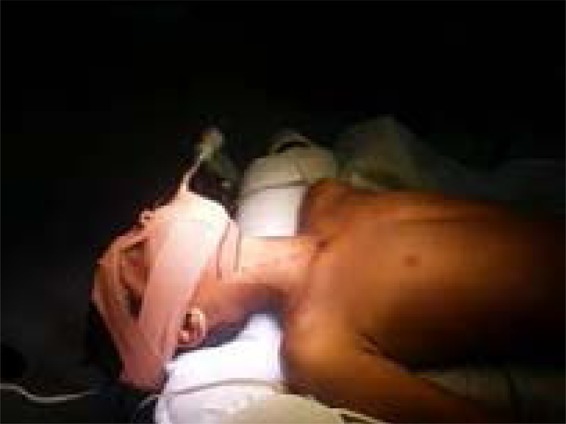
Patient positioning and skin incision for ACDF for C2-C3 fracture/subluxation.

**Fig. 5b:**
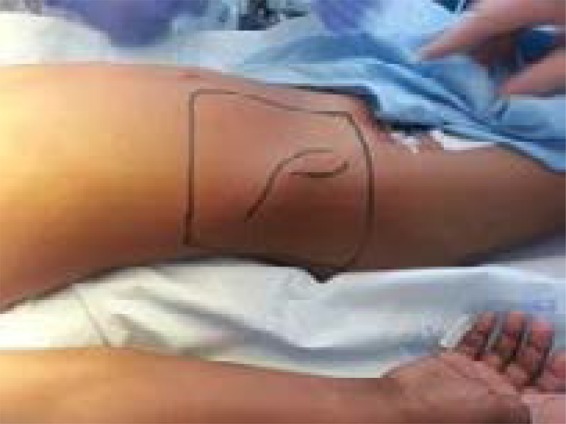
Skin incision for autologous iliac crest bone graft for ACDF.

**Fig. 6:**
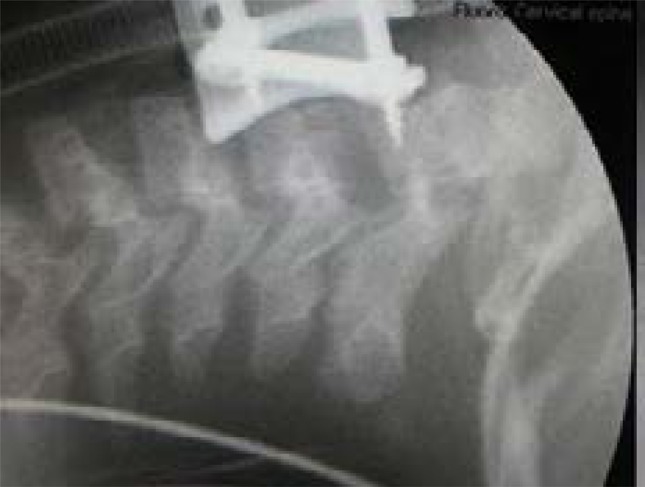
Intra-operative fluoroscopic guidance for anterior cervical plating after C2-C3 discectomy and fusion with autologous iliac crest bone graft.

**Fig. 7:**
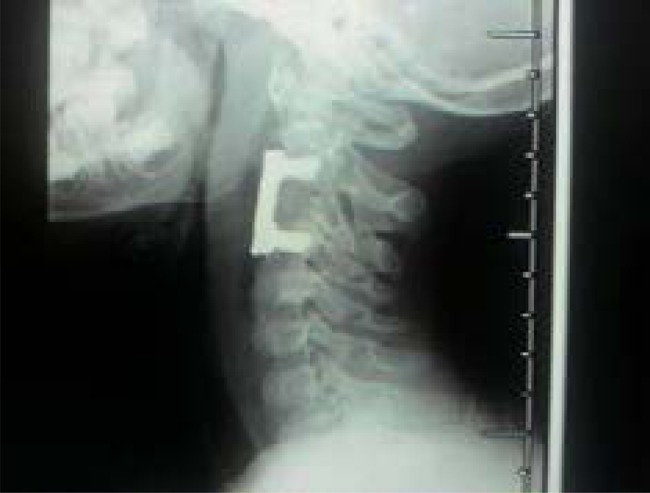
Post-operative lateral plain X-ray on the same patient after completion of the ACDF.

The intraoperative findings revealed an intact C2-C3 intervertebral disc which was, however, found to be separated from the C3 superior endplate through the cartilaginous layer. This probably was the factor in the unusual radiological appearance of the subluxation which showed the anteroinferior edge of C2 vertebra sitting on the superior surface of C3 vertebra with associated posterior displacement of the C2 vertebral body and its attached C2-C3 intervertebral disc into the spinal canal. Also there was clear evidence of acute injury and no signs of an old injury as evidenced by the absence of facet ankyloses and the relatively easy reduction. The soft cervical collar was continued post-operatively. The patient remained stable, recovered full motor power and was actively mobile post-surgery. He was subsequently discharged home on day 7 post surgery to the care of his Aunt and for outpatient follow up in the Paediatric Spinal Clinic as well as with Social Workers. It is of note that despite the radiological consideration of the C2 fracture as an old fracture, there was no history of any previous cervical spine trauma. However, a lot of social issues were uncovered during the patient's hospitalization necessitating the involvement of the hospital Social Workers in the care of the patient. The patient was discharged to the care of his Aunt, with no objection from the mother of the patient. A follow up plain cervical spine X-ray showed adequate reduction and satisfactory alignment of C2-C3 with the anterior cervical plate/screws in-situ (Fig. 7).

## Discussion

Injury at the upper cervical spine is a potentially fatal incident due to the risk of damage to the upper cervical spinal cord with resultant risk of respiratory paralysis. Such an injury is also a risk for other significant neurological disability. Our patient presented with unusually mild symptoms despite the degree of subluxation at C2-C3 with cord compression, thus reflecting the peculiarity of the paediatric cervical spine.

The paediatric developing spine has several peculiar features compared to the adult spine, and as well presents fundamentally different patterns of upper cervical spine injuries in children compared to adults.^10^,^11^ In children, the moderately large head forms a fulcrum of flexion at C2-C3 unlike in adult which is at C5-C6. Children also have elastic and lax ligamentous support and cartilaginous end plates, and relatively underdeveloped neck muscles, permitting greater range of movement under certain force. The vertebral bodies in children are wedge shaped anteriorly, the facet joints are aligned horizontally (most noticeable in the upper cervical spine) compared to the oblique alignment in adults.^8^–^11^ The horizontally aligned articulating facets, the increased ligamentous laxity and underdeveloped neck muscles make the bones of the paediatric cervical spine more mobile and less stable, and with a lower expectation of bony injury. Also injuries in the paediatric cervical spine would be expected to be at a higher level compared to cervical spine injuries in adults due to the higher point of flexion at C2-C3.^8^–^11^ Consequently, the management of such upper cervical spine injuries presents peculiar challenges concerning the use of external immobilization systems and surgical intervention especially when this occurs during the paediatric age where there is still significant potential for growth. In the paediatric age 0 to 8 years, the spine retains its immature features, becoming more like adult spine between the age of 9 to 16 years.^10^,^11^ However, with individual variation expected, it is believed that the cervical spine becomes more like that of the adult in structure and behaviour around the age of 8 and 9 years.^11^ Our patient being 8 years old falls within the transition between the immature features of the paediatric spine as well as the probable development into the adult spine, and therefore may be subject to injury pattern reflecting either of both. The upper cervical location of his injury is similar to the pattern expected in the younger paediatric age.

The cause of our patient's injury is a pedestrian vehicle accident and this is consistent with other studies which reports that the most common cause of upper paediatric cervical spine injury is motor vehicle accidents, as well as its association with head injury.^8^–^10^ In traumatic spondylolisthesis of the axis (TSA), hyperextension and axial load mechanism results in bilateral pars interarticularis fractures, and with the more severe injury patterns, rebound flexion or flexion/distraction mechanism results in disruption of the C2-C3 disc and posterior longitudinal ligament^1^,^2^,^13^ There may or may not be an associated anterior listhesis of the body of C2 on C3, which effectively enlarges the spinal canal. Hence despite significant fracture displacement, neurological damage is rare, due to decompression of the spinal canal by the separation of the fracture fragments.^1^,^2^,^14^ In contrast to the classic injury pattern in TSA, our patient had an associated separation of the C2-C3 intervertebral disc from the C3 vertebrae through the cartilaginous layer without any disruption of the C2-C3 intervertebral disc. This may be a factor of the lax cartilaginous end plate of the paediatric developing spine. Additionally, the wedge shaped anteroinferior edge of C2 (also a peculiarity of the paediatric developing spine) may have facilitated the posterior slippage of C2 on C3 probably from a flexion/distraction force. This may have led to the unusual position of the anteroinferior edge of C2 on the superior surface of C3 vertebra resulting in posterior displacement of the C2-C3 disc and narrowing of the spinal canal with cord compression (Fig. 2a). However, despite the narrowing of the spinal canal in our patient, there was no neurological fallout at presentation in the emergency resuscitation room. Although this seems to support the literature that neurologic disability related to paediatric cervical spine injury is uncommon if the child survives the initial impact of the trauma, it may not be unconnected with the relatively large intraspinal space at the upper cervical spine.^7^,^8^

The guidelines for the management of TSA is based on level III evidence and mainly aligns with Levine and Edwards classification of TSA.^1^,^2^,^15^ Recent literature reports opined that external immobilization is recommended as the initial management of traumatic spondylolisthesis of the axis, with surgical stabilization and fusion reserved for cases of severe angulation of C2 on C3, disruption of the C2-C3 disk space, and/or inability to achieve or maintain fracture alignment with external immobilization. 2,16 Non-operative management is considered the treatment of choice for stable TSA.^2^,^3^,^15^ In their recent study of TSA, van der Horst et al^1^ noted that the vast majority of patients with TSA can be managed conservatively with excellent functional outcomes, and concluded that non-surgical management is the default management for TSA. They elaborated that conservative management requires an initial period of skeletal traction followed by non-rigid immobilisation for stable injuries and rigid immobilisation for unstable injuries.^1^ The indications for surgery in their study were categorized into traditional indications for surgery, which results from failure of conservative management; and modern indications for surgery, which are based on specific ‘patient factors’ than with fracture configuration, displacement, angulation or perceived instability.^1^ The patient factors highlighted in their study included open head injuries, skull fractures/laceration, anticipated prolonged ventilation and some unique scenarios like psychosis, advanced pregnancy, and obesity that would make conservative management impractical. 1 However, in another recent study by Wei et al,^17^ they highlighted that despite the variously described surgical or nonsurgical treatments for Hangman's fracture, optimal treatment remains in question. They elaborated that although nonsurgical treatment were widely favoured in the primary treatment of TSA, healing was slow, uncertain, and treatment course was prolonged.^17^ They noted the complications of conservative treatment of Hangman's fracture to include 60% occurrence of anterior dislocation, C2-C3 angulation, pseudoarthrosis and recurrent axial pain.^15^,^17^ They also noted that there is a trend towards preferring surgical treatment as primary management for unstable Hangman's fracture due to the above complications in addition to associated disco-ligamentous injuries, the hope of improving neurological outcome or improving persistent axial pain suffered by patients after treatment with external orthosis.^15^,^17^ The aforementioned management issues in the reviewed literatures reflected in the challenges faced in the management of our patient. In reviewing our patient's injury, the unusual orientation of the C2-C3 subluxation in our patient with the associated C2-C3 disc involvement completely altered the injury pattern, rendering the segment unstable^2^ and significantly influencing optimal therapeutic choice for the patient. Hence it is quite likely that this may have resulted in more instability in the C2-C3 segment during the application of the Extension Halter traction, worsening the axial pain and causing more injury to the cord from the compressing C2-C3 disc. Definitely, for this paediatric patient surgical intervention would seem to be the preferred primary management.

There are different approaches/procedures to achieve surgical stabilization in TSA. The successful roles of anterior and posterior procedures for the surgical treatment of TSA when indicated, were highlighted by van der Horst et al^1^ and Wei et al^17^, although certain approaches may be favoured on a case to case basis.^1^ From their experience, van der Horst et al^1^ prefers the posterior approach using pedicle screws in C2 (and which may be used to lag the posterior arch to the body if necessary) and lateral mass screws in C3, with rods connected to them and on-lay bone graft for fusion.^1^ The technique is considered to be effective and safe and addresses both the C2 arch fracture and disco-ligamentous injury while allowing bony union.^1^ Wei et al^17^ agrees with the benefits of the posterior approach stating that the complex anatomic feature of the upper cervical spine makes the posterior approach preferred for its relative simple exposure with no major vascular or visceral structure.^17^ Also where the advantage of motion preservation in C2-C3 is desired, direct posterior fixation of the pedicles or the pars fracture with C2 lag screws solely across the fracture line has been performed.^2^,^17^,^18^ However, isolated C2 lag screw repair is no longer favoured on account of progressive anterolisthesis due to associated C2-C3 disco-ligamentous injury, failure to prevent kyphosis and loss of disc height including intraoperative neurological and vascular injuries with pedicle screw fixation.^1^,^17^,^18^ In their study, van der Horst et al^1^ demonstrated that C2-C3 posterior fusion provided better biomechanical stability than anterior C2-C3 plate, a finding supported by other biomechanical studies.^1^,^19^ In their recent case report of an unstable Hangman's fracture in an 80-year old male, Munakomi and Bhattarai^20^ also performed posterior fusion but opted for C1 and C3 lateral masses fusion, noting that it minimizes risk of vertebral artery injury and displacement of fractured segments into the canal by avoiding instrumentation in C2 entirely. They noted that the efficacy of their approach has been validated in the biomechanical study by Chittiboina et al.^21^ C1-C3 posterior instrumented fusion has also been performed for unstable TSA.^2^

Differing from the posterior approach; Li et al^22^ considered posterior approaches as not optimal, noting that they result in significant loss of C1–2 function, and that in a highly unstable Hangman's fracture, posterior fixation of C2–3 results in aggravation of the forward displacement of C-2 because of the intraoperative prone position, which may cause iatrogenic injury with extremely adverse consequences. In their study they used anterior C2–3 intervertebral disc excision or C-3 corpectomy, decompression and reduction, interbody implantation of an autologous iliac bone graft, and internal fixation with a titanium plate, and considered this approach to be a safe and effective procedure for the treatment of unstable hangman's fractures.^22^ They concluded that anterior discectomy/corpectomy and interbody fusion combined with internal fixation by plating is technically easy to perform and results in a relatively short length of fusion, and recommended that the anterior approach is especially suitable for hangman's fractures with intervertebral disc injury, which can result in spinal cord compression or spinal instability.^22^ Wei et al^17^ also expressed a preference for anterior C2–3 discectomy and fusion (ACDF) and noted that it is an effective strategy for unstable Hangman's fracture and supported by the literature.^23^ They accepted that although a high anterior exposure is considered to be complex and an unpopular technique due to difficulty in exposing the C2-C3 region,^24^ in their experience, surgical field exposures were without difficulty and there were no intraoperative complications during their procedures.^17^ From their experience of the 15 cases in their study, anterior approach offered satisfactory exposure for reduction of any C2-C3 displacement and fusion.^17^ They, however, noted that anterior cervical plate is inappropriate for patients with poor cervical vertebral bone quality, such as bone cyst, which could not provide sufficient pullout strength at the screw-bone interface. They therefore utilized cervical cage, specifically polyetherether ketone (PEEK material), without plating for C2–3 discectomy and fusion (ACDF) in management of type II/IIA Hangman'sfracture, which they noted required a less invasive approach and showed good clinical and radiological results. 17 Concerns raised about inadequate stability in the use of PEEK cage without plating for Hangman's fracture were answered by its complementary configuration to adjoining endplate surface, its retention teeth and bilateral titanium spikes on its surfaces, which they noted provides secure fixation and prevents migration/extrusion of the cage.^17^ In addition to other advantages noted in their use of PEEK cage without anterior plating, Wei et al^17^ also performed biomechanical evaluation of PEEK cage for type II Hangman's fracture, and this revealed no significant difference in range of motion (ROM) of lateral bending, rotation and extension between the cage group and bone graft plus plating group, except ROM of flexion, which was considered partly compensated by hard cervical collar.^17^,^25^ Caution was, however, emphasized not to apply PEEK cage solely for all cases of Hangman's fracture. Specifically, Wei et al^17^ recommended that PEEK cage solely should not be used to treat type III Hangman's fracture due to severe instability. In contrast to either anterior or posterior approach only, Xie et al^14^ in their study combined anterior C2-C3 reduction and fusion and posterior compressive C2 pedicle screw fixation for the management of unstable Hangman's fractures as single stage 360° fusion. They concluded that for Hangman's fractures with significant deformity and gapping, immediate single-stage anterior-posterior reduction, instrumentation, and arthrodesis achieve superior postoperative reduction and long-term functional outcomes.^14^

In our patient, the injury pattern did not fit into any of the classic four categories of TSA based on Levine and Edwards classification.^15^ However, it bears close characteristics to TSA Type IIa injuries which result from a distractive force with the neck in a hyper-flexed position, with associated significant angulation, no or minimal translation, and with damage to the inter-vertebral disc and the posterior longitudinal ligament thereby rendering them unstable.^1^–^3^ However, instead of anterior listhesis which is a common feature in TSA and which results in widening of the spinal canal, there was a posterior displacement of C2 on C3 in our patient, with resultant spinal canal stenosis and cord compression. An interesting observation in our patient is the initial interpretation of the CT findings by the radiologists, which considered the pars fractures as old C2 fractures, and this was based on the finding of minimal soft tissue signal. However, the Spinal Unit did not think this was the case because intraoperatively there was clear evidence of acute injury. Also we did not think it was an old fracture as there was no facet ankyloses and the C2-C3 reduction was relatively easy. Although the treatment of TSA varies depending on surgeon and institutional preferences, it is generally accepted to treat TSA type IIA by reduction in extension and use of halo vest but with avoidance of traction.^1^ However, the associated C2-C3 disc displacement rendered our patient's injury unstable and may not have responded to any form of conservative treatment with or without traction. The use of traction in our patient seemed to have worsened his pain and induced neurological deterioration possibly from cord injury from the disc compression. Surgical intervention was therefore the most appropriate option to treat this unique injury in the child. Posterior approach may not be an appropriate choice considering the displaced intervertebral disc with the spinal cord compression. Anterior approach offered the best chance of reduction of the posteriorly displaced C2 vertebra, spinal cord decompression through discectomy, and C2-C3 fusion. The standard anterior Smith-Robinson approach was used with a 4-hole anterior titanium plate/screws and autologous iliac crest bone graft fusion. Despite the high anterior cervical location, we found exposure without difficulty, consistent with the findings in the study by Wei et al.^17^

## Conclusion

Paediatric cervical spine injuries are uncommon, but present many potential pitfalls in management.^11^ A high index of suspicion for upper cervical spine injuries should be accorded to children who sustain head injuries from motor vehicle accidents or falls from height in view of the uniqueness of the paediatric cervical spine. The treatment of traumatic spondylolisthesis of the axis (TSA) should be individualized based on the injury characteristics. Surgical stabilization techniques via the anterior, posterior, and combined anteroposterior approaches have been described in the literature.^1^,^2^,^14^,^17^,^18^,^20^,^22^ Although this demonstrates the enormous challenges that may be encountered in decision making for the optimal treatment of unstable TSA, it nonetheless reveals the various therapeutic options available to surgeons in the management of unstable TSA in children. From our case report, anterior cervical discectomy and fusion even in children is a safe and effective procedure for unstable TSA or after failed conservative therapy in stable TSA.

## Abbreviations

TSA: Traumatic spondylolisthesis of the axis
PVA: Pedestrian vehicle accident
CT: Computed tomography
MRI: Magnetic resonance imaging
ACDF: Anterior cervical discectomy and fusion
C-spine: Cervical spine
